# Investigations into the Diversity and Distribution of tRNA and Phylogenetics of Translation Factors in Amoebozoa-Infecting *Nucleocytoviricota*

**DOI:** 10.3390/v17030328

**Published:** 2025-02-27

**Authors:** Thaís I. R. Moreira, João Victor R. P. Carvalho, Clécio A. C. Filho, Júlia W. Souza, Bruna L. de Azevedo, Jônatas S. Abrahão, Rodrigo A. L. Rodrigues

**Affiliations:** 1 Virus Laboratory, Department of Microbiology, Federal University of Minas Gerais (UFMG), Belo Horizonte 31270-901, MG, Brazil; thaisirmoreira@gmail.com (T.I.R.M.); jvrodrigues934@gmail.com (J.V.R.P.C.); clecioalonso@outlook.com (C.A.C.F.); jwinklersbio@gmail.com (J.W.S.); azvdobruna@gmail.com (B.L.d.A.); jonatas.abrahao@gmail.com (J.S.A.); 2 Retroviruses Laboratory, Department of Preventive Veterinary Medicine, School of Veterinary Medicine, Federal University of Minas Gerais (UFMG), Belo Horizonte 31270-901, MG, Brazil

**Keywords:** giant virus, Nucleocytoviricota, tRNA, translation factors, genomics, evolution, diversity

## Abstract

Translation is a sine qua non process for life as we know it. Translation factors (TFs) and tRNAs are rare among viruses but are commonly found in giant viruses of the class *Megaviricetes*. In this study, we explored the diversity and distribution of tRNAs in giant viruses that were isolated and replicated in amoebae (phylum Amoebozoa), and investigated the evolutionary history of TFs to gain insights into their origins in these viruses. We analyzed the genomes of 77 isolated giant viruses, 52 of which contained at least 1 tRNA. In most of these viruses, tRNA sequences are dispersed throughout the genome, except in Tupanviruses and Yasmineviruses, where most tRNAs are clustered in specific genomic islands. The tRNAs in giant viruses often contain introns, with 73.1% of the genomes exhibiting at least one intronic region in these genes. Codon usage bias (CUB) analysis of various giant viruses revealed at least two distinct patterns of codon preferences among closely related viruses. We did not observe a clear correlation between the presence of tRNAs and CUB in giant viruses. Due to the limited size of these genes, we could not confidently investigate their phylogenetic relationships. However, phylogenetic analysis of TFs found in giant viruses often position these viruses as sister groups or embedded between different eukaryotic taxa with high statistical support. Overall, our findings reinforce the complexity of key components of the translation apparatus in different members of *Nucleocytoviricota* isolated from different regions of Earth.

## 1. Introduction

Viruses are the most abundant and ubiquitous biological entities in nature. It is estimated that there are around 10^31^ viral particles distributed throughout the Earth that are able to parasitize organisms from all domains of life [1,2,3,4,5]. Defining viruses is not a trivial task, but they are traditionally conceived as obligate intracellular parasites and differ from their cellular hosts mainly because they lack genes involved in energy metabolism (e.g., enzymes of the citric acid cycle) and in protein translation, such as transfer RNAs (tRNAs), translation factors (TFs), and aminoacyl-tRNA synthetases (aaRS) [6,7]. The absence of these kinds of genes, among other factors, makes them dependent on the host cell machinery to replicate the genome and to synthesize proteins that are essential to viral progeny biosynthesis [8,9]. The lack of a translational gene-set in viruses is one of the main arguments to exclude them from a tree of life [10].

Transfer RNAs are molecules that play an important role in the protein biosynthesis since they carry amino acids to the ribosome and help in the decodification of messenger RNA (mRNA) information into amino acids [11,12,13]. Structurally, tRNAs are composed of a short chain that ranges from 70 to 100 nucleotides. They have a cloverleaf secondary structure that is composed by five elements: an acceptor arm, a D-arm, an anticodon arm, a TΨC arm (T-arm), and a variable arm [11,13]. Also, tRNAs present an L-shaped tertiary structure whose arms are formed due to the stacking of the acceptor arm and the T-arm and the stacking of the D-arm and the anticodon-arm [11,13,14]. These tRNAs components have different roles in protein translation. For example, the recognition of aaRS depends on the acceptor arm and on the D-arm. Aminoacyl-tRNA synthetases (aaRSs) are enzymes that catalyze the esterification of a tRNA to its cognate amino acid [15,16]. Additionally, the tRNA anticodon-arm is responsible for interaction with mRNA codon, which enables the elongation of the peptide chain by delivering the proper amino acid [11,12,17].

Although most viruses are completely dependent on host translation machinery, tRNA genes were described in some dsDNA viruses, such as bacteriophage T4, herpesviruses, chloroviruses, and baculoviruses [18,19,20,21]. Noteworthy, the presence of translation-related genes in viruses is remarkably described in viruses of the phylum *Nucleocytoviricota*, which comprises the so-called giant viruses [22]. Of the viruses included in this phylum, those belonging to the class *Megaviricetes*, order *Imitervirales*, harbor the vast majority of these elements, especially in the family *Mimiviridae* [23]. The first mimivirus isolate, Acanthamoeba polyphaga mimivirus (APMV), was described to code for six types of tRNAs, four different aaRS, and five translation factors, which astonished the scientific community [7]. After mimivirus discovery, other giant viruses were isolated, and their genomes exhibited a great abundance and diversity of translation apparatus components. Moumouviruses and megaviruses, the closest relatives of mimiviruses, encode up to seven types of aaRS, including all genes previously found in APMV [24]. Klosneuviruses genomes code for 25 tRNAs and 19 aaRS, while Tupanvirus and Yasminevirus code for up to 70 tRNAs and 20 types of aaRS, which is considered the most complete translational apparatus coded by a virus so far [23,25,26,27]. Thus, it is notable that the *Imitervirales* discovery was determinant to expand the known viral translational-related gene-set. Despite this, amoeba-infecting viruses of other taxa also have both tRNA and translation factors. These elements were already described for faustoviruses [28], marseilleviruses [29], medusaviruses [30], molliviruses [31], pandoraviruses [32], and pitho-like viruses [33], demonstrating the huge contribution of giant viruses in the expansion of the knowledge on viral translated-related genes. It is interesting to note that these genes are expressed during the viral replication, as observed for mimiviruses [34].

In addition to tRNA and aaRS, other translation factors are found in giant viruses related to the initiation, elongation, and release of the polypeptide chain during translation. These genes were first found in the APMV genome, which codes for three initiation factors, named initiation factor 4A and 4E (IF4A and IF4E) and initiation factor SUI1, in addition to one elongation factor eF-TU and a peptide chain release factor eRF1 [7]. Experimental evidence using gene silencing by siRNA suggests that IF4A is functional and necessary for mimiviruses to begin protein synthesis during the infection in *A. polyphaga* [35]. Computational and in vitro analysis evidenced that elongation and release factors found in mimiviruses and marseilleviruses are functional [36]. eRF1 in mimiviruses exhibits bacterial-like regulatory mechanisms, even though it is a eukaryotic/archaeal homologous gene, which raises important questions about the origin and evolution of the translation-related apparatus in giant viruses [37]. Indeed, this is a hot topic, revolving around giant viruses since the discovery of APMV, with a few studies suggesting that giant viruses are reminiscent of a fourth domain of life and coexisted with cellular ancestors [38,39,40,41]. In contrast, other studies, including different lineages of mimiviruses, pandoraviruses, and klosneuviruses, suggest that giant viruses originated from smaller viruses, evolving in an accordion-like model of gene gain and loss throughout evolutionary history [25,42,43,44]. Revisiting this intriguing aspect of giant viruses, including the most recent groups of giant viruses, especially considering the high-quality genome assemblies available in public databases, can help us to better understand the origin of the translation apparatus in *Nucleocytoviricota*.

The discovery of giant viruses highlighted the fact that viral genomes can be more complex than generally expected and that they code for genes that were never or rarely described in other viruses before, such as the translation apparatus genes. However, the knowledge about giant viruses’ tRNAs genes and translation factors are still not widely explored, requiring constant updates. Furthermore, ongoing efforts to isolate new giant viruses add more layers of complexity when it comes to the abundance, diversity, and evolution of viral tRNA and translation factors. In this context, revisiting old paradigms and considering new data may bring us closer to a better understanding of the nature of these genes in viruses and how they are related to cellular organisms. In this work, we analyze the diversity, distribution, and prevalence of tRNAs coded by different amoeba-infecting giant virus groups. We compare general characteristics that are found in each tRNA, such as sequence sizes, GC content, the presence of introns, and their location in viral genomes. In addition, we investigated the phylogenetic relationship of different translation factors found in giant viruses, based on new data originating from new isolates uncovered in recent years, updating the phylogenomic information of these genes in the phylum *Nucleocytoviricota* by considering the most recent findings. This work expands our knowledge of important components of the translation apparatus found in giant viruses of amoebae and puts us a step closer to better understanding the nature of one of the most complex groups in the virosphere.

## 2. Materials and Methods

### 2.1. Dataset Construction

A comprehensive literature search was conducted to identify the isolates of giant viruses. Complete genomes from various amoeba-infecting giant virus isolates were manually retrieved from GenBank in FASTA format (sequences available until 31 December 2023). The viral groups analyzed included different isolates of Yasminevirus [27], Tupanvirus [26], Mimivirus [7], Moumouvirus [45], Megavirus [46], Kaumoebavirus [47], Pacmanvirus [48], Faustovirus [28], Marseillevirus [49], Orpheovirus [50], Cedratvirus [51], Pithovirus [52], Pandoravirus [32], Mollivirus [31], Medusavirus [30], Fadolivirus [53], and Clandestinovirus [54] (Table S1). For Clandestinovirus, Yasminevirus, and Pandoravirus mammoth, two deposits were found in the database; therefore, both were considered for the analyses. The viral sequences accessed in this work are registered under the SISGEN (Brazilian national system for the management of genetic heritage and associated traditional knowledge) access codes ADBF241 and A2291C9.

### 2.2. tRNAs Prediction

The set of genome sequences was analyzed to predict tRNA genes using the ARAGORN software v.1.2.41 with the following parameters: type: tRNA; allow intron: yes; topology: linear or circular (depending on the virus); strand: both [55]. Only the genomes for which ARAGORN predicted at least one tRNA were selected for subsequent analyses. In cases where the isotype and anticodon predicted by ARAGORN were not accurately discriminated (indicating two isotypes for the same tRNA gene, called ambiguous tRNA), an additional prediction was performed using tRNA-scanSE 2.0 [56]. Genomes that were not predicted to have any tRNA genes or that presented ambiguous and/or indeterminate tRNA were excluded from the study (See Table S1).

### 2.3. Analyses of tRNAs Sequences

tRNAs GC-content and the presence and size of introns were obtained using ARAGORN [55]. Viral tRNA gene data and information were recorded in spreadsheets using Microsoft Excel 2022 (Microsoft Corporation, Redmond, WA, USA), and the dataset was plotted and analyzed using GraphPad Prism 9.0 (GraphPad Software Inc., San Diego, CA, USA).

To analyze the gene position in the genome and check for possible tRNA gene clusters, we selected a representative from each viral group meeting the condition of tRNA > 1. For representatives of the *Megamimivirinae* subfamily, we selected one for *Mimivirus*, *Moumouvirus*, and *Megavirus* genus, as well as both Tupanviruses with available genomes. tRNA genes were considered clustered if they had a tRNA gene density > 1 within 500 bp. The PROKSEE server (https://proksee.ca/projects, accessed on 4 March 2024) was used to compare and graphically represent the data [57].

### 2.4. Codon Usage Analysis

The codon usage bias of each virus was calculated from each virus reference genome using the online calculator provided by Dr. Jamie McGowan (https://jamiemcgowan.ie/bioinf/codon_usage.html, accessed on 21 July 2024). The data were compiled with tRNA presence information and plotted using GraphPad Prism 9.5.

### 2.5. Multiple Sequence Alignment

The tRNA gene sequences were aligned using Muscle software with standard parameters, implemented in MEGA 11 [58,59]. Genes with the presence of introns had these regions removed from the sequence predicted by ARAGORN. The tRNA-leucine gene was used to display the alignment between the nucleotide sequences, and a representative was selected from each viral group that met the condition of having at least 1 tRNA-leucine gene.

### 2.6. Phylogenetic Analysis of Translation Factors

Using a modified version of the pipeline described by Yutin et al. (2014), we analyzed the amino acid sequences associated with the translational gene-set of *Tupanvirus altamarinense*, *Marseillevirus massiliense* (isolate Marseillevirus marseillevirus), Pithovirus LCPAC404, *Yasminevirus saudimassiliense* (isolate GU-2018), *Alphaorpheovirus massiliense* (isolate IHUMI-LCC2), and *Mimivirus bradfordmassiliense* (isolate APMV). The query sequences used are listed in Table S2. Briefly, we compared each protein against the NCBI nr and RefSeq databases using an E-value cutoff of 0.01 and a composition-based statistic for compositional adjustments in BLASTp [60]. For the nr database, we selected the top 250 hits, and for RefSeq, 5000 (or all hits if the limit was not reached). These data were combined into a custom database and searched again using the same query proteins but without compositional adjustments [42]. Hits were then divided into 3 datasets and subjected to a redundancy removal algorithm (BLASTClust): (i) the top 20 hits clustered with 95% identity, (ii) the following 500 hits clustered with 75% identity, and (iii) the remainder clustered with 65% identity. The remaining dataset was aligned using MUSCLE [57] and trimmed in trimAL [61] with a gap threshold of 30%. Phylogenetic reconstructions were performed using the IQ-TREE 2 software [62] based on the maximum likelihood method. The substitution models and site rate heterogeneity were selected automatically using MODEL FINDER [63], and statistical support for the nodes was evaluated using bootstrap values from 1000 replicates using UFBoot2 [64]. The resulting trees were visualized and edited using iTOL [65].

### 2.7. Data Availability

All raw sequence datasets used for phylogenetic constructions, as well as the alignments and the tree files in Newick format, can be accessed at FigShare: https://dx.doi.org/10.6084/m9.figshare.27115387 (accessed on 21 July 2024).

## 3. Results and Discussion

### 3.1. tRNA Diversity and Distribution in Amoebae Giant Viruses

**Figure 1:**
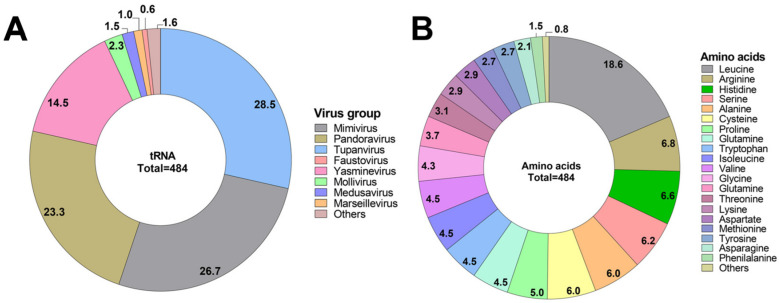
Diversity and abundance of tRNA in amoeba-infecting giant viruses. (**A**) Distribution of predicted tRNA percentage for each viral group analyzed. Mimivirus, moumouviruses, and megaviruses are merged in Mimivirus group; ‘Others’ refers to cedratviruses (*n* = 3), Orpheovirus (*n* = 2), and Clandestinovirus (*n* = 1). Since these were small values, we merged them so that they would be easy to see in the chart. (**B**) Amino acid prevalence among the predicted tRNAs.

We obtained a total of 77 complete genomes of giant virus isolates until the end of 2023 from GenBank, distributed as follows: 17 Faustoviruses (22.1%), 11 Mimiviruses (14.3%), 10 Pandoraviruses (13.0%), 8 Marseilleviruses (10.4%), 6 megaviruses and cedratviruses (7.8% each), 5 Moumouviruses (6.57%), 2 Kaumoebaviruses, Molliviruses, Pacmanviruses, Tupanviruses, and Medusaviruses (2.6% each), and 1 Clandestinovirus, Orpheovirus, Yasminevirus, and Pithovirus (1.3% each). Among the 77 genomes retrieved from GenBank, 52 had at least 1 tRNA. We predicted a total of 484 tRNAs, representing a diversity of 61 of the 64 anticodons described for the 22 different amino acids [66], in addition to the detection of one stop-tRNA in Yasminevirus. By comparing the analyzed viral groups, we observe that the total number of tRNAs ranged from 1 to 138. Together, the two isolates of Tupanviruses had the highest tRNA number among the viral groups, with 138 tRNAs (138/484 = 28.5%), followed by mimiviruses (including moumouviruses and megaviruses) isolates with 129 tRNAs (129/484 = 26.7%) and Pandoraviruses with 113 tRNAs (1137/484 = 23.3%) (Figure 1A). The high number of tRNAs found in the family *Mimiviridae* is in accordance with what was previously described [22,23,25]. Furthermore, even with a single isolate in analysis, Yasminevirus contributed 70 tRNAs (70/484 = 14.5%). On the other hand, the groups that expressed a lower number of tRNAs were Faustovirus and Cedratviruses (3/484 = 0.62%), followed by Pacmanvirus and Orpheovirus with two tRNAs each (2/484 = 0.41%) and Clandestinovirus with only one predicted tRNA (1/484 = 0.2%) (Figure 1A).

Although the family *Mimiviridae* is known for coding the most complete viral translation gene apparatus [22], it is noteworthy that the number of isolates of a viral group can influence the number of predicted tRNAs. For example, the number of mimivirus isolates analyzed here (*n* = 24; including members of the family *Mimiviridae*), which is a group with a high number of tRNAs, is bigger than the number of isolates in groups with a smaller number of tRNAs, such as Orpheovirus, Cedratvirus, and Pacmanvirus with one or two sequences analyzed (Table S1). On the other hand, the Faustoviruses group presented a small number of tRNAs compared with mimiviruses, but a substantial number of Faustovirus isolates were analyzed (*n* = 17). It suggests that a less complete set of tRNAs is characteristic of this group. Furthermore, these elements are rarely found in isolated Marseilleviruses, but interestingly they were observed in uncultured relatives [67,68]. It highlights the importance of keeping efforts to isolate new viruses around the world, as it can help to improve our knowledge about giant viruses’ translation gene-set.

The number of tRNAs considering the cognate amino acid varied from 90 to 1 (non-canonical tRNAs). tRNA-leucine was the most prevalent type with 90 registered occurrences (18.6%) (Figure 1B). On the other hand, the non-canonical tRNAs, selenocysteine and pyrrolysine [11], together with the stop tRNA, had the lowest prevalence, with only two tRNAs (Pyl-tRNA) (0.4%) and one Sec-tRNA (0.2%). Previous works that analyzed bacteriophages concluded that the number of genes coding for tRNA was related to genome size [69,70]. We noticed similar correlation considering the genomes of giant viruses isolated using amoebae (Table S1). In our analysis, we found three major outliers in this correlation, namely the two Tupanviruses isolates (*Megamimivirinae*) and Yasminevirus (*Klosneuvirinae*), which have over 70 tRNA genes each, a number >4-fold higher compared to other giant viruses.

**Figure 2:**
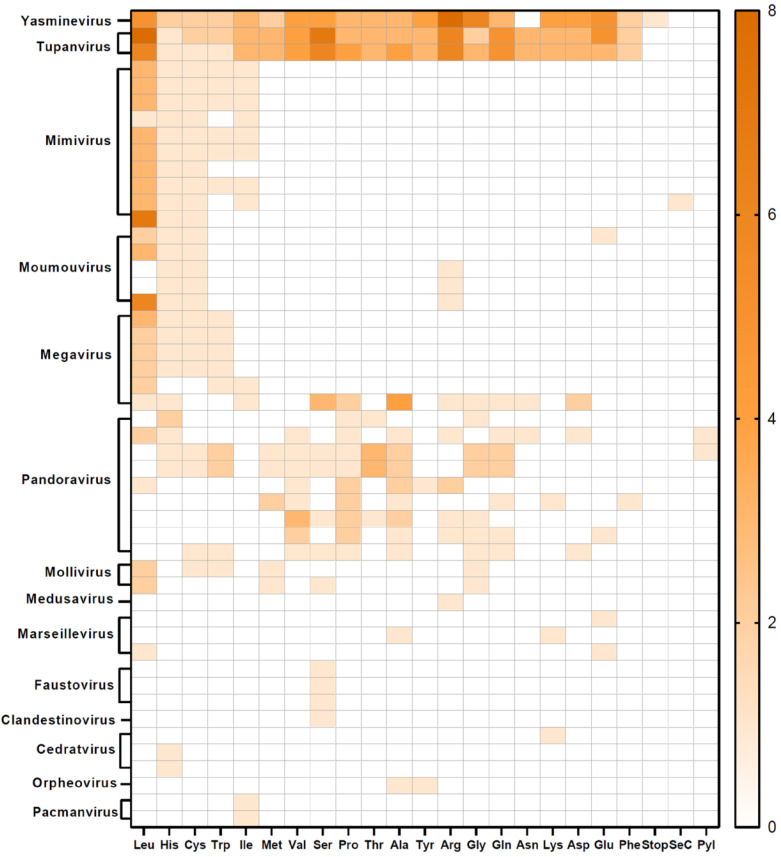
Individual distribution of tRNA cognate amino acids in giant viruses. The number of encoded tRNAs is depicted by color density. Blank spaces indicate the absence of tRNA in the corresponding viral genome. Forty-eight viruses were included, distributed in fourteen virus groups.

The less prevalent tRNA genes were found, respectively, in Mimivirus reunion Queen, Pandoravirus japonicus, and Yasminevirus saudimassiliense. When considering tRNA diversity, Yasminevirus and Tupanvirus are the groups with the higher diversity, with Yasminevirus coding tRNAs for 19 different amino acids and 1 stop-tRNA, while Tupanviruses have tRNA genes for all the 20 canonical amino acids (Figure 2). In addition, when analyzing different isolates from the same viral group, Mimiviruses had a low variation in the amino acids’ prevalence, while Pandoraviruses were the group with the higher variation in tRNA types, exhibiting no clear pattern of tRNA distribution (Figure 2).

**Figure 3:**
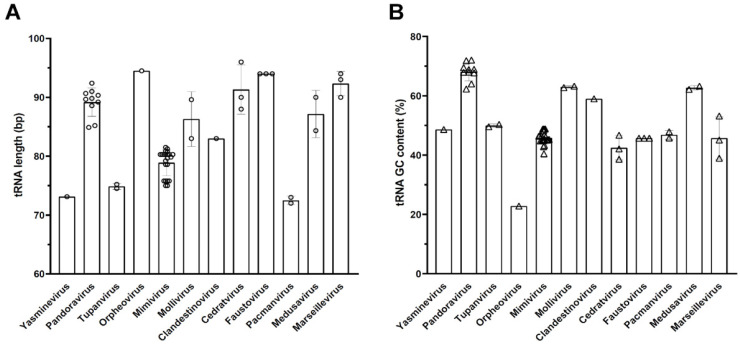
Analysis of tRNA in giant viruses of amoebae. (**A**) Average tRNA size per viral group; (**B**) Average GC content of tRNA per viral group. Mimivirus, moumouvirus, and megavirus are merged. Each symbol in the graphs (open circle in **A** and open triangle in **B**) represents data related to one virus isolate.

The size of tRNAs varied individually from 69 nt (tRNA-stop of Yasminevirus saudimassiliense) to 101 nt (tRNA-leucine of Pandoravirus japonicus), and the GC content of these tRNAs ranged from 13% (tRNA-tyrosine of Orpheovirus IHUMI-LCC2) to 84% (tRNA-proline of Pandoravirus dulcis). Assessing by groups, the average tRNA size ranged from 72.5 nt (Yasminevirus) to 94.5 nt (Orpheovirus), and the GC content ranged from 22.8% (Orpheovirus) to 68.1% (Pandoravirus). Among the virus groups analyzed, Orpheovirus had the highest average tRNA size with 94.5 nt (Figure 3A), while the average GC content was the lowest among all groups, with a value of 22.8% (Figure 3B). Faustovirus and Marseillevirus had average sizes close to Orpheovirus, 94 and 92.3 nt, respectively, and the average GC content was also close between the two groups, 45.7% and 45.8%, respectively. Other groups that were close in size were Pandoravirus (89.6 nt), which also had the highest average GC content among the groups (68.1%), Cedratvirus (89 nt), but with the second lowest GC content (42.7%), Medusavirus (87.2 nt), and Mollivirus (86.3 nt), whose %GC was similar between the last two, 63% and 62.7%, respectively. Compared to the GC content of complete genomes, the tRNA GC content did not show major differences. The groups that showed a difference of more than 10% in relation to the GC content of the genomes for the tRNA were, in descending order, the Tupanviruses (20.7%), followed by the Mimiviruses (19.1%), Clandestinoviruses (15.5%), and Pacmanviruses (13.5%).

In general, tRNAs vary in length between 70 and 100 nucleotides [11], and this was also observed in the tRNA of the viruses included in this study. However, there were three tRNAs with a length out from the pattern, including a tRNA-leucine gene with 101 nt in length from Pandoravirus and a stop-tRNA gene (TTA anticodon) with a length of 69 nt in Yasminevirus. The presence of a stop tRNA in Yasmineviruses was predicted by ARAGORN and confirmed by tRNA-scanSE 2.0 in our analysis, and these data corroborate the previous description of Yasminevirus tRNAs [26]. The occurrence of this gene is atypical because it has not previously been described for other viruses or even for cellular organisms. Interestingly, the presence of this same tRNA was found in a virophage, and it was hypothesized to be indicative of stop codon reassignment in the hosts of this virophage [71]. Therefore, further studies are necessary to confirm the nature of this stop tRNA, ruling out the possibility of a false positive result from the algorithm. In the case of a positive outcome, additional analyses are needed to confirm the role of these genes during the replication cycle of these viruses.

### 3.2. tRNA of Giant Viruses Are Populated by Introns

**Figure 4:**
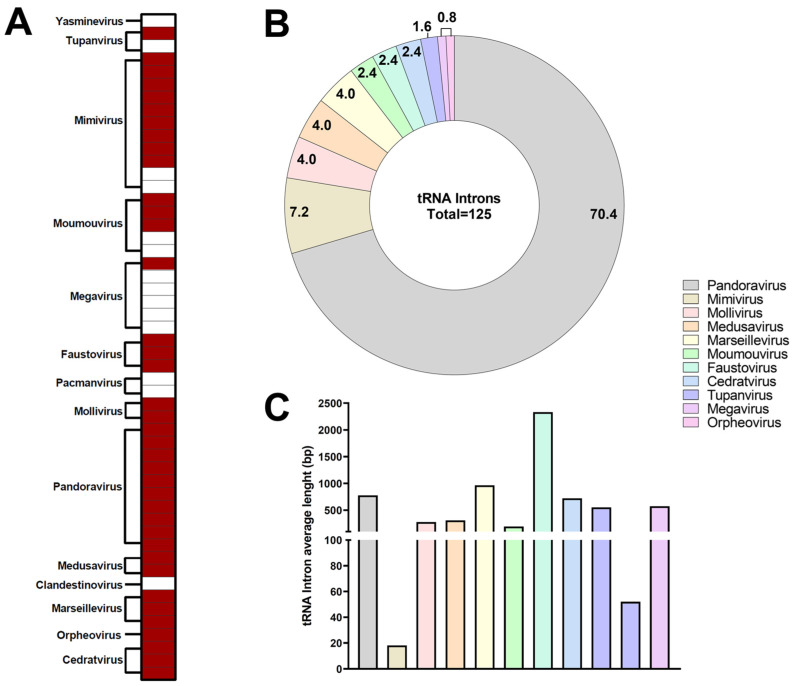
Presence of Introns in Giant Viruses tRNA. (**A**) Presence of introns in tRNA in the viruses of the study groups (*n* = 38); (**B**) Distribution of the incidence of introns in the viral groups; (**C**) Average size of the introns within the viral groups.

We observed the presence of introns in the tRNA sequence in most of the genomes included in this study (38/52 = 73.1%) (Figure 4A). Among these 38, 10 are pandoraviruses (26.3%), 9 are Mimiviruses (23.7%), 3 moumouviruses, Marseilleviruses, cedratviruses, and Faustoviruses (7.9% each), 2 Molliviruses and Medusaviruses, (5.3% each), and 1 Tupanvirus, megavirus and Orpheovirus (2.6% each). The group with the highest incidence of tRNA-containing intron insertions was Pandoravirus, totaling 88 introns (70.4%), which was highly discrepant from the second group, Mimivirus, with only 9 (7.2%). The Molliviruses, Marseilleviruses, and Medusaviruses had the same result of 5 tRNAs-containing introns (4.0%), followed by moumouviruses, Faustoviruses and cedratviruses with 3 (2.4%), Tupanviruses with 2 (1.6%), and Orpheoviruses with only 1 (0.8%) (Figure 4B). Faustoviruses had the highest average size of introns, with the same size in their three tRNAs (2332 nt) (Figure 4C). On the other hand, the Marseillevirus group showed a high discrepancy with the first group, even in the average intron size value of 964 nt, ranging from 120 nt to 1480 nt. Introns in *Marseilleviridae* have been recently described in Marseillevirus cajuinense and other isolates, not included in this study, thus reinforcing our findings [67]. A similar variation occurs in Medusavirus (37 nt to 1383 nt) and Tupanviruses (6 nt to 1096 nt). The intron sizes in Mimiviruses tRNA are the shortest among all evaluated viruses, with ~20 nt in length.

The number and position of the introns in the tRNA vary depending on the organism [72]. Most giant viruses have been able to exceed the known range of 6 to 133 nt for eukaryotic introns, showing an average of 584 nt. Several baculoviral tRNA genes also exhibited long introns, unlike chloroviruses that follow the known interval pattern [20,21]. In Mimivirus, all introns were inserted at canonical positions 37/38. It interrupts the structure of the anticodon, splitting the tRNA into non-functional halves and making splicing essential [73]. The other viruses had the introns inserted more in non-canonical positions between 27/28 and 41/42. These positions do not seem to interrupt the general structure of the tRNA. The introns found in eukaryotic and archaeal tRNA genes are mainly inserted at position 37/38, also known as the canonical position of enzymatically spliced introns in tRNA precursors, but they can also be found inserted at non-canonical positions, with archaea having a greater diversity of intron insertion positions compared to eukaryotes [73,74]. In addition, both can present more than one intronic sequence in the same tRNA gene, a characteristic that was not observed in any virus having an intronic sequence. The origin of tRNA introns in the canonical position is considered ancestral, and positional variability reflects changes in enzyme specificity for splicing, presumably the result of coevolution among introns and these enzymes [72,73,74,75].

Moreover, it was possible to observe the presence of possible gene duplication for some sequences in groups of viruses that have kept the fragments completely conserved. Micheli et al. (2022) infer that the association of introns with gene duplication increases the stability of the duplicated copies, and both processes are evolutionarily supported [76]. At the same time, it is curious that long introns show none or few changes in the sequence since they do not directly interfere with the function of the gene, as they are non-coding genes and tend to accumulate more mutations. Little is known about the destination and function of tRNA introns, but a selective advantage in transporting these introns within the genome is possible and deserves further investigation [77].

The occurrence of introns has also been reported in other genes of giant viruses, such as in the main capsid protein in Faustoviruses (with up to 18 introns present in Faustovirus S17) and Mimiviruses [28,78,79,80]. Introns have also been detected in other giant amoeba virus genes, such as the DNA polymerase gene [32]. The presence of introns suggests the existence of an influence that increases gene expression in eukaryotes, and the presence of splicing described in other viruses may reinforce this hypothesis of increased gene expression.

### 3.3. Location of tRNA in Giant Viruses’ Genomes

**Figure 5:**
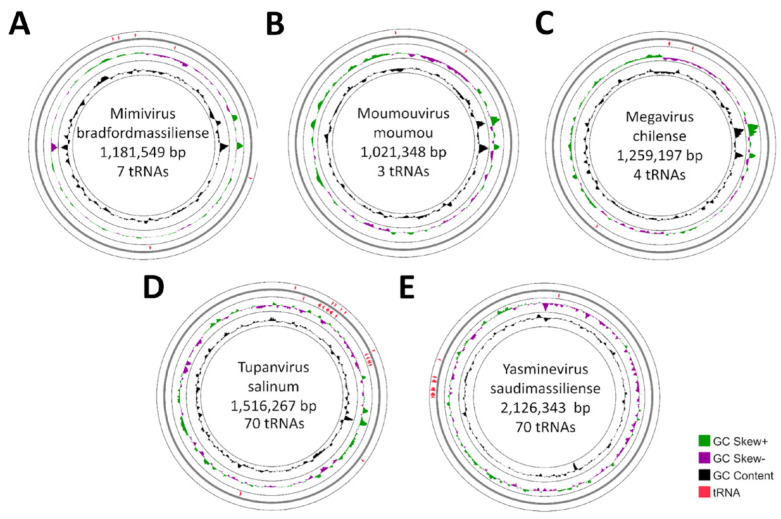
Distribution of tRNA in viral genomes of *Mimiviridae* representatives. Graphical circular map of the genome of the components of the viral groups. In the center: the name of the virus, genome size, and amount of tRNA. From the outside to center: ring 1 shows the tRNA genes on the direct strand and ring 2 on the reverse strand, ring 3 shows the G + C% content, and ring 4 shows the GC deviation. (**A**) Mimivirus; (**B**) Moumouvirus; (**C**) Megavirus; (**D**) Tupanvirus; (**E**) Yasminevirus.

To assess the position of the tRNA gene in the viral genome, one representative from each viral group that fulfills the condition of having more than one tRNA was selected. Therefore, the Cedratvirus and Faustovirus groups were not represented in this analysis. Most of the tRNA was dispersed throughout the viral genomes. Some viruses exhibited completely dispersed tRNA, including Orpheovirus IHUMI-LCC2, Marseillevirus marseillevirus, Pandoravirus salinus, Mollivirus sibericum, and Medusavirus medusae (Figure S1). Others, such as Mimivirus bradfordmassiliense (Figure 5A) and Moumouvirus moumou (Figure 5B), showed only one region with two tRNAs in tandem, with a difference of 1 to 47 nt between the end of one tRNA and the beginning of the other. The others were also dispersed throughout the genome. The two isolates from the Mimivirus group had the same tRNA located in this region, a histidine tRNA and a cysteine tRNA. However, in the case of Moumouvirus moumou, this region was close to ±96 Mbp in the genome, while for Mimivirus bradfordmassiliense it was close to ±350 Mbp in the genome. The Megavirus chilense (Figure 5C) showed only one region with two tRNA-leucines relatively close together (1054 nt), while the others were completely dispersed.

The dispersion of tRNA throughout the genome changes for viruses that encoded a high number of these genes, such as Tupanvirus salinum (Figure 5D), where approximately 96% are clustered and 97.2% of the total tRNA located in the first quarter of the genome, and Yasminevirus saudimassiliense (Figure 5E), with approximately 97% of its tRNA found in tandem and 98.6% located in the last quarter of the genome. Tupanvirus salinum had only three tRNA isolated in the genome. The others were in groups of 2 to 8 tRNA, with a spacing of 2066 to 301,070 bp between the end of one group and the beginning of another. In addition, some types of tRNA present in a number >1 in the genome tended to be in the same region as their peers, as noticed for tyrosine, phenylalanine, and methionine. It was also observed that some types of tRNA appeared to be associated with others, such as glutamine and serine, which were grouped together in two different genomic regions. Yasminevirus saudimassiliense had only two tRNA isolated in the genome. The others were in groups of 2 to 10, with the spacing between the end of a grouping and the beginning of the other ranging from 904 to 18,047 bp. These groups were found near their similar ones, between approximately 1494 kbp and 1564 kbp of the genome. They also had some types of tRNA encoded in the same region as their peers, such as proline, cysteine, and isoleucine. As in Tupanvirus, glutamine and serine were also located in the same genomic region in Yasminevirus.

Morgado et al. (2019) argued that viruses with smaller genomes tend to organize their tRNA in clusters in favor of genome compaction [70]. According to this idea, giant viruses would not have the same tendency, and these genes would be dispersed throughout the genome. Unlike the positive correlation between the length of the genome and the number of tRNAs, the clustering of these genes occurs inversely to the genome size. The same study also reported an exception in the Tupanviruses, which carry 10 to 11 genes in small groups, while the other giant viruses had their tRNA genes dispersed homogeneously throughout the genome. Our observations are consistent with the previous study, but it was observed that Tupanviruses carry up to eight genes in clusters, with the highest incidence being between four and six genes. Yasmineviruses, isolated in 2016, were also found to have tRNA organized into clusters, similar to Tupanviruses. In addition, Duncan et al. (2020) indicated that, except for viruses in the family *Mimiviridae*, most large DNA viruses code only a few or no genes for tRNA, and when a virus encodes at least two tRNAs, they are not clustered, emphasizing the findings of our study [20]. However, it is important to note that except for Tupanvirus and Yasminevirus, which had several genes clustered together, other viruses in the *Mimiviridae* family had only one cluster with two tRNAs, with the majority of the tRNA genes scattered throughout the genome. Therefore, the proportion of dispersed genes is higher than those organized in clusters within the group of giant amoeba viruses.

### 3.4. Codon Usage Bias and tRNA in Giant Viruses

Studies indicate that the presence of tRNA-coding sequences in DNA virus genomes is linked to the need for these parasites to adjust translational capacity during the infection process, either as a response to the host immune activity or to complement the available tRNAs due to differences in codon usage patterns [81]. Using bacteriophage T4 as a model, Miller et al. (2003) showed that, despite possessing eight tRNA sequences in its genome, these products do not have a positive correlation with the most abundant amino acids in the viral proteome, even though the related codons have a higher frequency in T4 genes than in those of the host [19]. In this context, by calculating the codon usage bias (CUB) of several amoeba-infecting viruses, we aimed to compare the CUB profile and analyze if there is any correlation between the presence of viral tRNAs and the codon preferences of the genes of these giant viruses.

**Figure 6:**
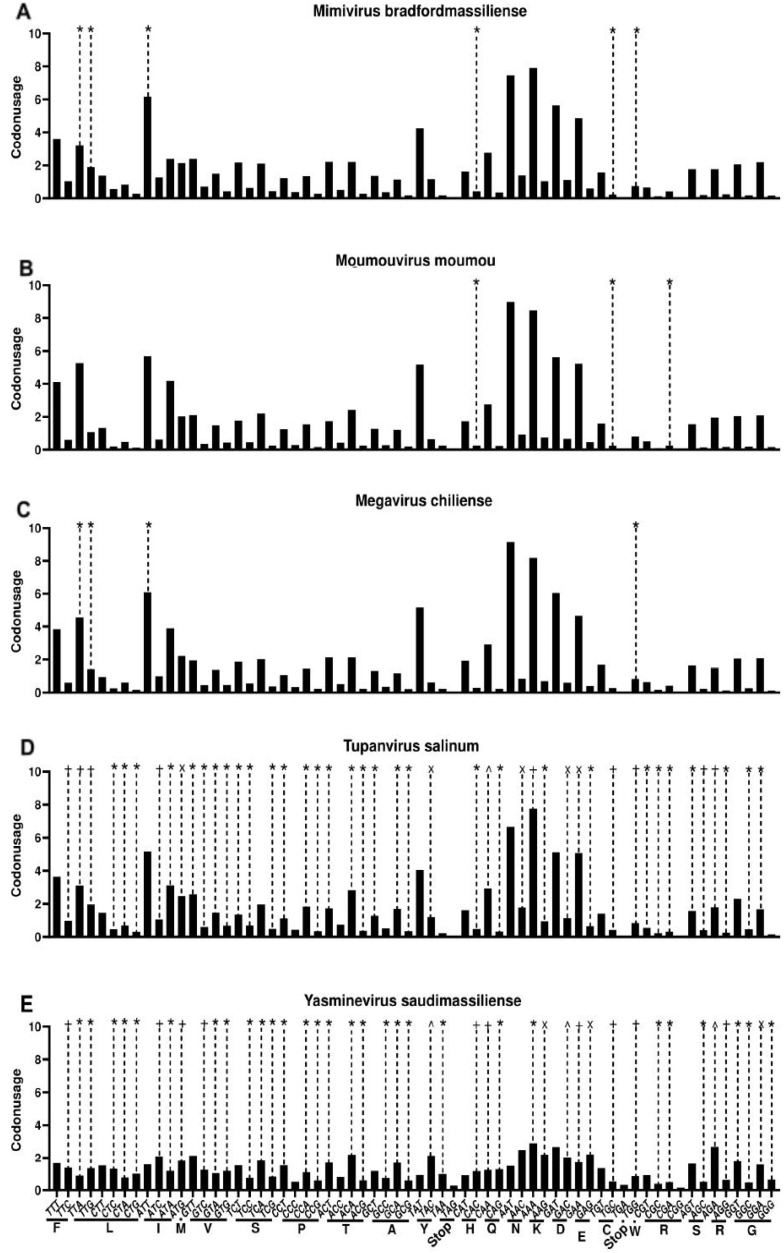
Codon usage of representatives of different genera of the family *Mimiviridae*. (**A**) *Mimivirus*; (**B**) *Moumouvirus*; (**C**) *Megavirus*; (**D**) *Tupanvirus*; (**E**) *Klosneuvirus.* Dashed lines indicate the presence of a tRNA related to the corresponding codon. Codon usage is represented as percentage of use. Corresponding amino acids are indicated below the codons. The symbols above each tRNA bar represent the number of this tRNA found in the reference genome of each virus; *—one tRNA, +—two tRNAs, x—three tRNAS, ^—four tRNAs.

Members of the family *Mimiviridae* (order *Imitervirales*) exhibited two distinct patterns of CUB, wherein mimiviruses (genus *Mimivirus*), moumouviruses (genus *Moumouvirus*), megaviruses (genus *Megavirus*), and Tupanviruses (genus *Tupanvirus*) have very similar profile of codon usage, with strong preference for A-T-rich codons (Figure 6A–D). These data reinforce what was observed years ago for mimiviruses [82]. Curiously, Yasminevirus (genus *Klosneuvirus*) exhibits a different profile of CUB, with more homogeneous usage of A-T and G-C rich codons (Figure 6E). Klosneuviruses have important genomic differences compared to mimiviruses and Tupanviruses, resulting in two different subfamilies, i.e., *Klosneuviridae* and *Megamimivirinae* [83]. These genomic and evolutionary distances can be related to the distinct CUB patterns observed in this study. In addition, Yasminevirus infects *Vermamoeba vermiformis* [27], while mimiviruses infect *Acanthamoeba* sp. [84], and such a distinct CUB pattern can be related to the host differences among these viruses. Notwithstanding, Tupanviruses can also replicate in *V. vermiformis* [85], which suggests that the CUB pattern observed here may not be directly related to the viral host. Additional evidence in favor of this is that Orpheovirus, an ovoid-shaped virus belonging to the proposed family “Orpheoviridae” (order *Pimascovirales*), exhibits a very similar CUB pattern compared to *Megamimivirinae* viruses (Figure S2A), even though it replicates in *V. vermiformis* [86,87]. Marseilleviruses exhibit a more uniform CUB profile, even though it is possible to observe a slight preference for A-T-rich codons (Figure S2B). In contrast, molliviruses, pandoraviruses, and medusaviruses prefer G-T-rich codons (Figure S2C–E).

It is interesting to note that, beyond the apparent phylogenetic relationship when comparing the CUBs of the analyzed viruses, the GC content of the genomes may be related to the compartment where genome replication occurs. There is evidence suggesting that viruses that require the host nucleus for this process have a higher GC content (and thus require more G-C-rich codons) than those independent of the nuclear compartment [88]. Mimiviruses, Marseilleviruses, and Orpheovirus replicate the genomes in their viral factories within the host cytoplasm, while other viruses, like medusaviruses, seem to depend on the amoeba nucleus for this process [30,49,87,89]. A more in-depth analysis merging virus replication and codon usage patterns will be important to improve our comprehension of the mysteries of giant viruses-host co-evolution.

Finally, no positive correlation was found between the codons they are associated with and their necessity for each virus, with viruses possessing tRNAs for both highly and lowly used codons (Figure 6 and Figure S2). The patterns of Tupanvirus and Yasminevirus significantly diverge from the rest of the studied viruses due to the high number of encoded tRNAs, but no correlation to which codons these tRNAs bind was found.

### 3.5. Phylogenetics of Translation Components of Giant Viruses

The origin of viral tRNAs remains unclear and is a significant challenge to uncover. However, like many other analyzed viral genes, they might have been acquired through horizontal gene transfer (HGT) from various sources, such as their host, bacteria, and other viral species that infect the same host [20,21]. Evidence of this putative acquisition may be observed in Tupanviruses, which are phylogenetically close to mimiviruses but infect a broader range of hosts, exhibiting a more robust arsenal of tRNA genes [26]. As evidenced here, there is no clear pattern in the distribution of tRNA among the giant viruses that could allow a strong hypothesis about their origin. The most plausible scenario is that each group of viruses, i.e., different taxa of giant viruses, acquired the tRNA independently throughout history. The most prevalent gene is the tRNA-leucine, found in 27 of the 48 viruses in the study. The sequences for the gene encoding tRNA-leucine were very diverse among the viruses analyzed, with only 2 conserved sites (99 and 107) for all 30 sequences, indicating high divergence in these genes, even considering the anticodon region (Figure S3). Widmann et al. (2010) describe tRNAs as unfavorable candidates for phylogenetic studies due to the small size of the sequences [90]. To this end, some studies use concatenated genes, in which the individual tRNA sequences are connected in a single alignment to produce larger sequences. However, using concatenated genes can induce errors in the inference of their origin [91,92,93]. Furthermore, using individual tRNA sequences with introns can lead to errors in phylogenetic reconstructions, as they accumulate more mutations, resulting in high rates of divergence between the sequences [94].

**Figure 7:**
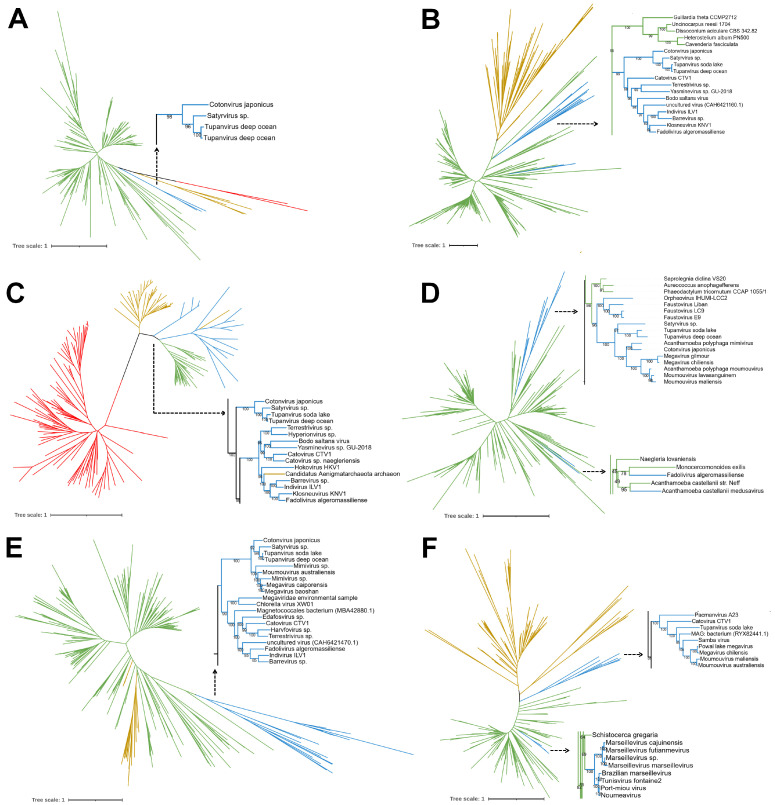
Phylogenetic reconstruction of translation factors found in giant amoeba viruses. A different number of sequences (*n*) were included in each analysis upon availability in Genbank and removal of redundancies. (**A**) Initiation factor 2 alpha subunit (*n* = 183); (**B**) Initiation factor 2 beta subunit (*n* = 365); (**C**) Initiation factor 2 gamma subunit (*n* = 194); (**D**) Suppressor of initiator codon mutations 1 (*n* = 230); (**E**) Mimivirus GTP-binding translation elongation/initiation factor (*n* = 302); (**F**) Eukaryotic release factor 1 (*n* = 234). The scale bar indicates the number of substitutions per site. Branches containing giant viruses are highlighted as subset trees. Trees were built considering 1000 bootstrap replicates. Groups are colored in blue (Viruses); red (Bacteria); yellow (Archaea); and green (Eukarya).

Although we have conducted an extensive analysis of the presence and dispersion of tRNAs in giant viruses, we also sought to analyze the essential factors for the translation process to occur. To do this, we searched for the initiation, elongation, and termination elements in different amoeba-infecting *Nucleocytoviricota* (Table S2) and used their respective amino acid sequences to search for homologs in the viruses selected in this work to perform phylogenetic reconstructions for each element. In this way, from 11 datasets, we were able to generate 6 trees with a reasonable resolution to gain insights and discuss the position of these genes in giant viruses of amoebae among cellular organisms (Figure 7). Since we were unable to establish a reliable outgroup given the purpose of the analysis, we drew unrooted trees and avoided any assumptions about the evolution of these genes among giant viruses.

#### 3.5.1. Translation Initiation Factor 2 Alpha Subunit

The eukaryotic translation initiation factor 2 (eIF2) is a heterotrimeric protein composed of the alpha, beta, and gamma subunits. Its main function is to initiate translation by delivering the methionyl-tRNA complex to the 40s ribosomal subunit [95]. Consequently, its alpha subunit (eIF2α) has a regulatory role, with the phosphorylation of its Ser51 residue down-regulating total protein synthesis [96]. In this dataset of 183 protein sequences (including 4 *Megamimivirinae* sequences), we observed that the viral homolog of eIF2α appears as a sister group of eukaryotes (Figure 7A). It is important to cite that this gene was found only in this group of viruses and in a few representatives, which can happen due to a limitation of the database or a natural case. This is similar to what was previously reported for these genes found in klosneuviruses [25], thus reinforcing the hypothesis of origin of these genes among giant amoeba viruses through HGT events from eukaryotic organisms. Curiously, the presence of distant homologues of eIF2α has been described in poxviruses and iridoviruses [97,98]. We constructed a new tree including sequences from these large nucleocytoviruses and observed very long branches given the high divergence in the sequences, thus providing a low-confidence phylogenetic reconstruction (Figure S4). This can be explained by the fact that these genes seem to share a conserved structure with eukaryotic eIF2alpha, a typical case of molecular mimicry, or in this case, structural mimicry, but they may have a completely different origin [97]. If we assume these genes as true homologues, the sequences have undergone a high degree of modification but have conserved protein folds. Another more plausible scenario is to consider this as an evolutionary convergence event, where genes from different origins have similar folds and may exhibit similar functions. The discovery and characterization of new giant viruses carrying this gene will be crucial to improving our understanding of the origin and evolution of this translation factor in the virosphere.3.5.2. Translation Initiation Factor 2 Beta.

Following the eIF2 subunits, the beta subunit (eIF2β) is responsible for binding to mRNA, using its lysine residues and C2-C2 motif [99]. The phylogenomic reconstruction of eIF2β shows the presence of giant viruses among eukaryotes divided into two distant branches. A major branch with high bootstrap support (99) comprises most of the giant viruses that we included in the analysis, suggesting that this gene could already be found in the ancestor of the family *Mimiviridae*. Two other sequences were found distantly from mimiviruses and klosneuviruses and belong to genomes recovered from metagenomes, which are Marseillevirus LCMAC103 and Pithovirus LCPAC404 [68]. We did not find any homologues among Marseilleviruses or Pithoviruses’ isolates, so the lack of data on these groups of viruses may explain their position in the phylogenetic tree. However, based on this result, we can speculate that different groups of giant viruses may have obtained this gene from different sources at different times during evolution. Our tree is consistent with others previously published [25,42], with archaea remaining as an outgroup and giant viruses putatively acquiring the gene from eukaryotes at least twice during evolution.

#### 3.5.2. Translation Initiation Factor 2 Gamma Subunit

The eIF2 gamma (eIF2ɣ) is the last subunit of eIF2, with a heterotrimer core responsible for binding to methionyl-tRNA and the guanine nucleotide, functioning as the docking site for GTP/GDP [95]. Notably, we found viral homologs of this gene only in members of the class *Imitervirales* (Figure 7C). Our results are similar to those observed when considering only klosneuviruses, where they appear as a sister group to eukaryotes [25]. Now, with more giant virus sequences available, we observe that the putative origin of this gene in *Nucleocytoviricota* is much more complex than previously thought. We observed a more distant branch composed of cotonvirus, satyrvirus, and tupanviruses and another clade composed of klosneuviruses, both with high bootstrap support (100). Furthermore, it should be noted that bacteria do not possess the canonical eIF2ɣ; they encode the selenocysteine-specific translation elongation factor, which is related to the archaeal/eukaryotic translation factor analyzed here [100]. This high divergence is observed in our tree, with Bacteria forming a very distant branch from the others. Interestingly, an archaeal sequence, related to an assembled metagenomic genome of “Candidatus Aenigmataarcheota”, is found within one of the viral groups, even with the presence of other archaea in the tree, and is more similar to viral sequences than to archaeal ones. Further investigation of this genome could reveal interesting new features shared between archaea and giant viruses or correct a misidentified sequence in the database.

#### 3.5.3. Suppressor of Initiator Codon Mutations 1

The suppressor of initiator codon mutations 1 (SUI1) is a translation initiation factor found in yeasts that functions with eIF2 and methionyl-tRNA in directing the ribosome to the translation start site [101]. Here we observed giant viruses deeply related to eukaryotes, which suggests that events of HGT could be the source of this gene among giant viruses (Figure 7D). Interestingly, different large and giant viruses appear as a monophyletic group with high bootstrap support (96), a group that includes orpheovirus, faustoviruses, and many representatives of the family *Mimiviridae.* Despite using different datasets, our results are similar to those observed by Marcelino et al., where some giant viruses appear as sister group of eukaryotes. Curiously, two viral genes are found inside another eukaryotic group. It is a sequence from fadolivirus, a *Mimiviridae* that infects *Vermamoeba vermiformis* and a medusavirus that infects *Acanthamoeba castellanii* [30,60]. Interestingly, in this tree, the medusavirus shares a common ancestor with *A. castellanii*, encouraging the hypothesis of gene acquisition by its host, which can be better explored in future studies. Even when long branches were removed from the analysis, its sequence was still found at this position, so that the closest sequences are also from protozoa, suggesting that this may not be a methodological error. Thus, our finding indicates that this gene, as well as other translation-related genes, may have appeared in *Nucleocytoviricota* independently as lineage-related events, although some viruses may have an ancestral relationship. Yutin et al. observed a similar scenario a decade ago, when fewer isolates were available [42]. This reinforces the importance of continued efforts to isolate and characterize new giant viruses so that we can improve and gain more confidence in interpreting the mysteries surrounding the origin and evolution of these genes in the virosphere.

#### 3.5.4. Mimivirus GTP-Binding Translation Elongation/Initiation Factor

The mimivirus GTP-binding translation elongation/initiation factors are GTPase homologs to the eukaryotic GTP-binding protein 1 (GTPBP1), facilitating the delivery of amino acid-charged tRNAs to the A site of the ribosome [36]. Our phylogenetic reconstruction (Figure 7E) shows that this gene is ancient in the natural history of mimivirids, or else very divergent, since all the viruses sampled group together as a sister group of an eukarya-archaea branch. This gene is exclusive to this group of viruses, since others, such as pimascovirales, encode genes with similar functions but different origins [36]. Our results differ from those previously observed in other studies, probably due to the sampling strategy to construct the phylogeny dataset. Yutin et al. observed that these genes present in Marseilleviruses were closely related to eukaryotes (Amoebozoa), while Schulz et al. observed that this gene was possibly acquired by giant viruses in two different ways, both from eukaryotes [25,42]. In further studies, robust analysis focusing on this particular gene will improve our understanding of its putative origin and evolution among giant viruses.

#### 3.5.5. Eukaryotic Release Factor 1

The eukaryotic release factor 1 is responsible for recognizing stop codons and inducing the hydrolyzation of the ester bond linking the peptide chain and the peptidyl-tRNA complex in the P-site of the ribosome, facilitating the translation termination process [102]. Our analysis indicates that the homologs of this gene found in the giant viruses are closely related to eukaryotes, divided into two monophyletic branches, one containing *Mimiviridae* relatives and Pacmanvirus A23 and the other formed by the Marseilleviruses (Figure 7F). This pattern is found for almost all evaluated translation factors and in other elements not included in this study, with giant viruses possibly being sister groups of eukaryotes [103,104]. Marseilleviruses appear to have acquired these genes more recently from other eukaryotes, although more in-depth studies are needed focusing on investigating the origin of these genes. Our data are similar to those observed by Schulz et al., pointing to *Megaviricetes* as a sister group of eukaryotes, but Marseilleviruses are embedded in the cellular domain branch [25]. These results are slightly in contrast to that observed a decade ago by Yutin et al., possibly due to the sampling strategy used to construct the dataset [42]. Nevertheless, it is important to note that even when using different strategies to construct the trees, in all cases the Archaea appeared as an outgroup and the *Megaviricetes* as external to the eukaryotes, thus suggesting the presence of these genes among *Nucleocytoviricota* prior the irradiation of the Eukarya.

## 4. Conclusions

About twenty years ago, the scientific community was astonished by mimivirus’ giant and complex genome [7]. Over the last two decades, dozens of giant viruses infecting amoebae have been isolated, revealing biological and genomic features previously considered unimaginable in the virosphere, including numerous genes related to the translation process. Furthermore, in the past five years, metagenomic-based studies have demonstrated that the diversity and impact of giant viruses on our planet are far more significant than previously thought and understanding them is a challenge that is still far from complete [105,106]. The importance of metagenomics for the field of giant viruses is undeniable. Nevertheless, genomic analyses using isolated viruses offer the opportunity to gain insights into the biology and evolution of these isolates, which can be further investigated in vitro.

In this context, we explored 77 complete genomes of different groups of isolated giant viruses to delve into the diversity, distribution, and evolution of components of their translation apparatus—a gene-set that is quite rare in the virosphere but surprisingly common among giant viruses. Among the 77 genomes analyzed, 52 contained at least 1 tRNA, with Tupanvirus soda lake and Yasminevirus exhibiting the highest number and diversity of genes. tRNAs are most frequently found in members of the *Mimiviridae* family, followed by Pandoraviruses. We observed a direct correlation between genome size and the number of tRNA genes. However, Tupanviruses and Yasminevirus exhibited an excess of tRNA, approximately four times higher than any other isolate, even considering viruses with larger genomes, such as Pandoraviruses. Many predicted tRNAs contained introns, especially among Pandoraviruses. In most genomes, tRNA genes are dispersed, except in Tupanviruses and Yasminevirus. It is possible that the high copy number of tRNA in these viruses originated from gene duplication, establishing a tandem organization of tRNA [44]. Confidently defining the origin and evolution of genes involved in central metabolic processes, such as translation-related genes, remains a major challenge due to the heterogeneity of these genes among different realms of life. The identification and characterization of new giant viruses in the coming years will be of pivotal importance to enhance our understanding of the origin and biological impact of the viral translation machinery.

## Data Availability

No new data were created for this study. All genomic data used in this study are publicly available at the GenBank database. Accession numbers are available at Table S1. All raw sequence datasets using for phylogenetic constructions, as well as the alignments, and the tree files in Newick format can be accessed at FigShare: https://dx.doi.org/10.6084/m9.figshare.27115387.

## Supplementary Materials

The following supporting information can be downloaded at: https://www.mdpi.com/article/10.3390/v17030328/s1, Figure S1: Distribution of tRNA in giant viruses’ representatives. Graphical circular map of the genome of the components of the viral groups. In the center: the name of the virus, genome size and amount of tRNA. From the outside to center: ring 1 shows the tRNA genes on the direct strand and ring 2 on the reverse strand; ring 3 shows the G + C% content, and ring 4 shows the GC deviation. (A) Orpheovirus; (B) Marseillevirus; (C) Mollivirus; (D) Pandoravirus; (E) Medusavirus; Figure S2: Codon usage of representatives of different groups of amoebae-infecting giant viruses. (A) Orpheovirus; (B) Marseillevirus; (C) Mollivirus; (D) Pandoravirus; (E) Medusavirus. Dashed lines indicate the presence of a tRNA related to the corresponding codon. The symbols above each tRNA bar represent the number of this tRNA found in the reference genome of each virus; *—one tRNA, +—two tRNAs; Figure S3: Multiple sequence alignment of tRNA-Leucine in giant viruses; Figure S4: Phylogenetic reconstruction eukaryotic Initiation Factor 2 alpha subunit including sequences from poxviruses and iridoviruses. (A) Unrooted tree; (B) Mid-point rooted tree. The scale bar indicates the number of substitutions per site. Trees were built considering 1000 bootstrap replicates. Groups are colored in blue (Viruses); red (Bacteria); yellow (Archaea); and green (Eukarya). Table S1: General genomic features of giant viruses included in the study; Table S2: Translation-related proteins of amoeba-infecting *Nucleocytoviricota* used to construct the phylogenetic trees.
