# Supplementary material for: Investigations into the Diversity and Distribution of tRNA and Phylogenetics of Translation Factors in Amoebozoa-Infecting Nucleocytoviricota
Source: Viruses. 2025 Feb 27;17(3):328. doi: 10.3390/v17030328 (PMC11946776; doi:10.3390/v17030328)
Supplement: Supplementary file 1 [file viruses-17-00328-s001.zip › Table S2.pdf]

Supplementary Table S2. Translation-related proteins of amoeba-infecting *Nucleocytoviricota* used to construct the phylogenetic trees.

Protein	Virus	*Acession #
translation initiation factor 2 alpha subunit	Tupanvirus altamarinense	YP_010781204
translation initiation factor 2 beta subunit	Tupanvirus altamarinense	YP_010780625
	Yasminevirus GU-2018	VBB17804
	Pithovirus LCPAC404	QBK93402
translation initiation factor 2 gamma subunit	Tupanvirus altamarinense	YP_010780055
	Yasminevirus GU-2018	VBB18038
	Marseillevirus massiliense	YP_003406906
translation initiation factor 4e, 1	Tupanvirus altamarinense	YP_010780342
translation initiation factor 4e, 2	Tupanvirus altamarinense	YP_010780844
translation initiation factor 5A	Tupanvirus altamarinense	YP_010780185
translation factor SUI1-like 2	Tupanvirus altamarinense	YP_010780781
	Mimivirus saudimassiliense	YP_003986971
	Alphaorpheovirus IHUMI-LCC2	YP_009448830
translation initiation factor 4a	Tupanvirus altamarinense	YP_010780764
mimivirus GTP-binding translation elongation/initiation factor	Tupanvirus altamarinense	YP_010780064
	Yasminevirus GU-2018[a]	VBB17972
elongation factor aef-2	Tupanvirus altamarinense	YP_010781262
peptide chain release factor eRF1	Tupanvirus altamarinense	YP_010781258
	Marseillevirus massiliense	YP_003406874

*The accession number refers to the GenBank database.
[a]This sequence is not in the alignment, since it was removed during the redundancy analysis.
