# Supplementary figures and images for: Investigations into the Diversity and Distribution of tRNA and Phylogenetics of Translation Factors in Amoebozoa-Infecting Nucleocytoviricota
Source: Viruses. 2025 Feb 27;17(3):328. doi: 10.3390/v17030328 (PMC11946776; doi:10.3390/v17030328)

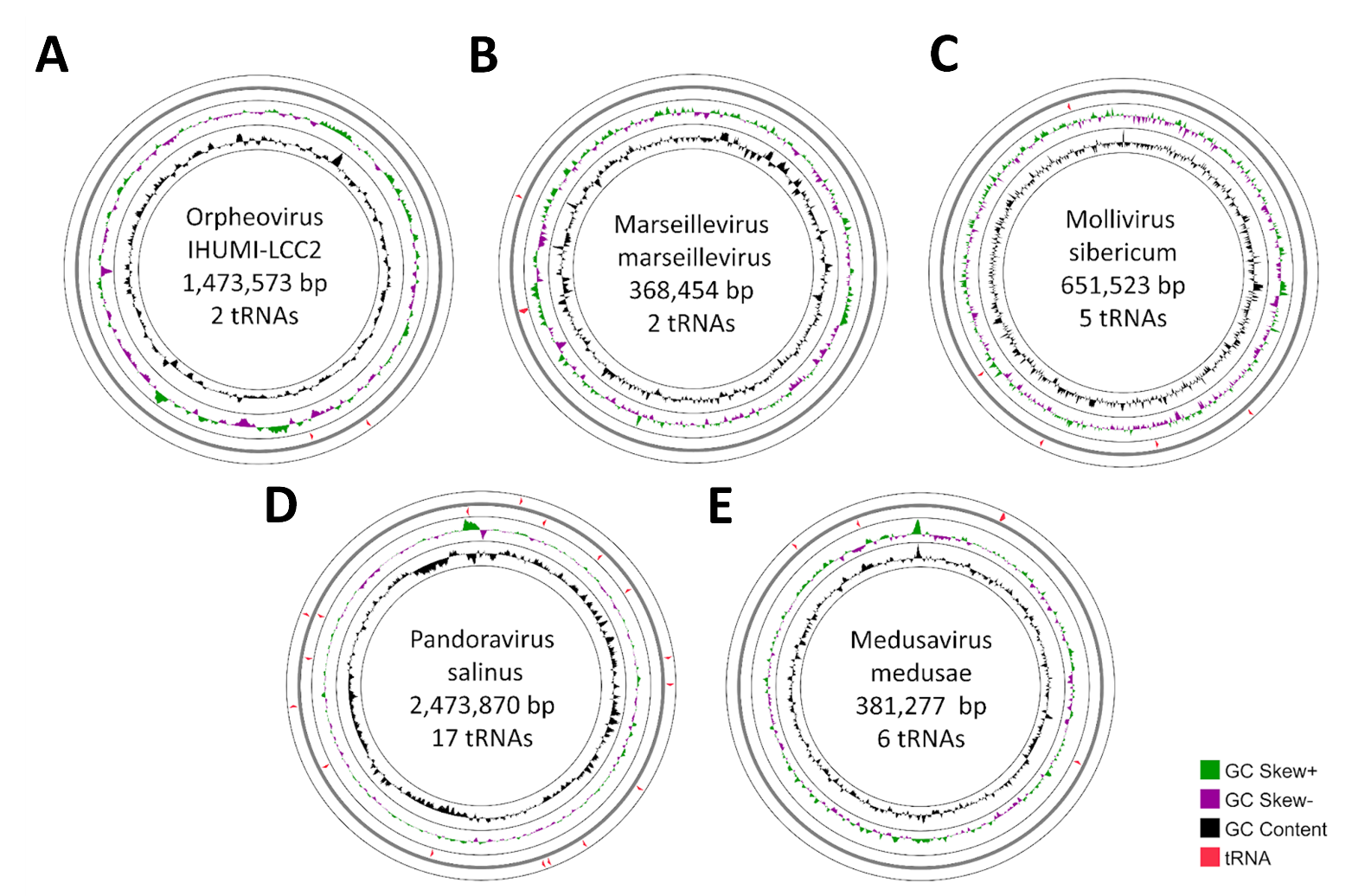

Supplement: Supplementary file 1 [file viruses-17-00328-s001.zip › Figure S1.tif]

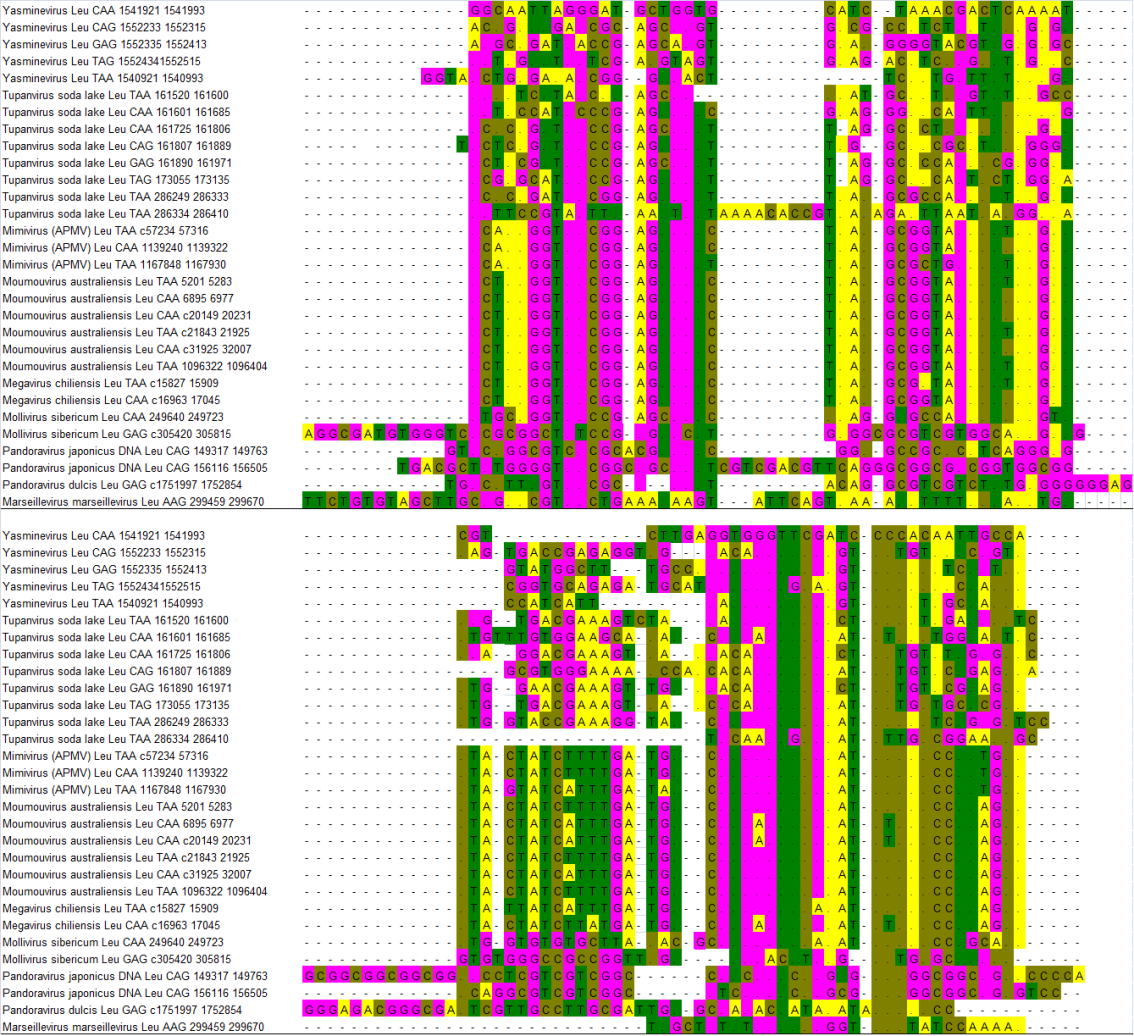

Supplement: Supplementary file 1 [file viruses-17-00328-s001.zip › Figure S3.tif]

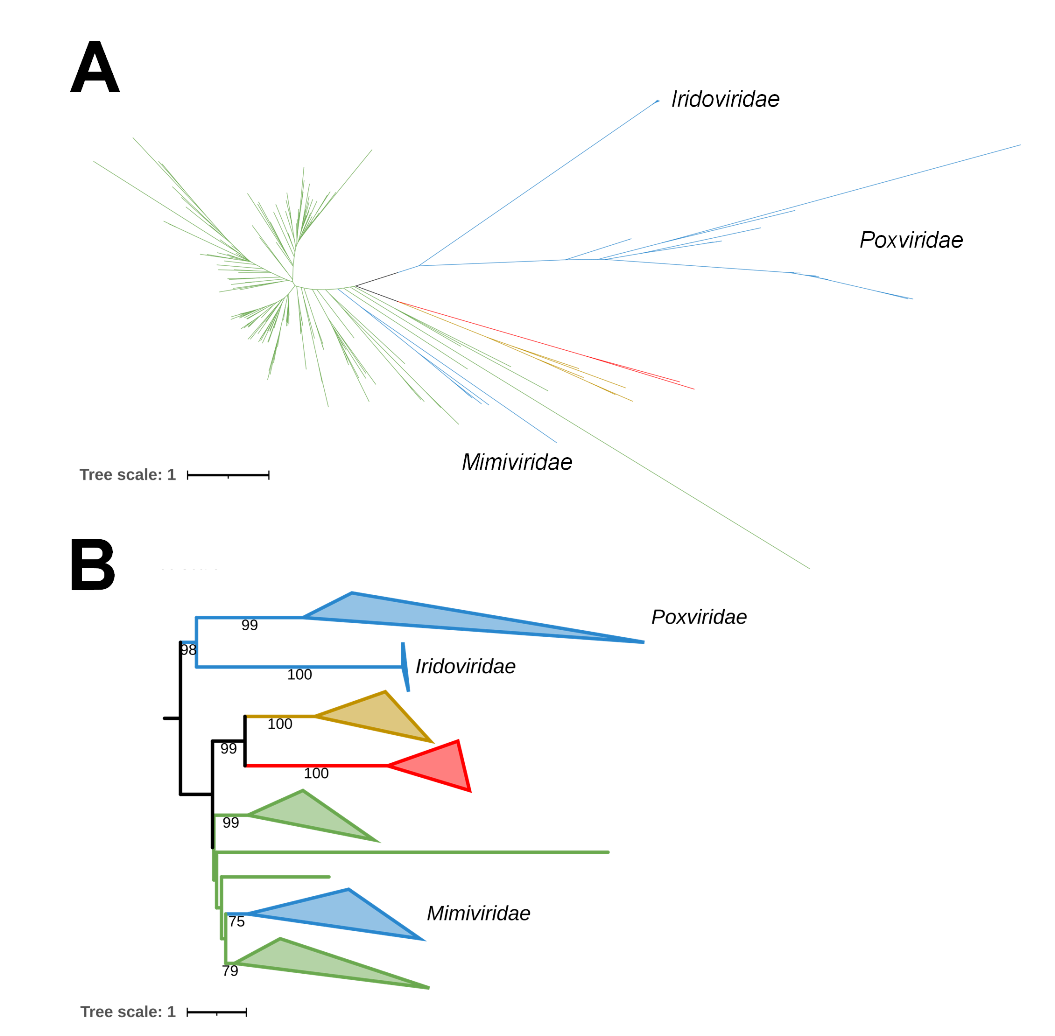

Supplement: Supplementary file 1 [file viruses-17-00328-s001.zip › Figure S4.tif]
